# Bacterial Diversity, Organic Acid, and Flavor Analysis of Dacha and Ercha Fermented Grains of Fen Flavor Baijiu

**DOI:** 10.3389/fmicb.2021.769290

**Published:** 2022-01-04

**Authors:** Yu’ang Xue, Fengxian Tang, Wenchao Cai, Xinxin Zhao, Wen Song, Ji’an Zhong, Zhongjun Liu, Zhuang Guo, Chunhui Shan

**Affiliations:** ^1^School of Food Science, Shihezi University, Shihezi, China; ^2^School of Food Science and Chemical Engineering, Hubei University of Arts and Science, Xiangyang, China; ^3^Xiangyang Liquor Brewing Biotechnology and Application Enterprise-University Joint Innovation Center, Xiangyang, China; ^4^Xiangyang Fen-Flavor Baijiu Biotechnology Key Laboratory, Xiangyang, China

**Keywords:** fermented grains, bacterial community, high-throughput sequencing, electronic senses, organic acid, Fen flavor Baijiu

## Abstract

Fen flavor Baijiu needs two rounds of fermentation, which will obtain Dacha after initial fermentation and Ercha after secondary fermentation. The quality of Baijiu is closely related to the microbes within fermented grains. However, the bacterial diversity in Dacha and Ercha fermented grains of Fen flavor Baijiu has not been reported. In the present study, the structure and diversity of bacteria communities within fermented grains of Fen flavor Baijiu were analyzed and evaluated using MiSeq platform’s HTS with a sequencing target of the V3-V4 region of the 16S rRNA gene. Through the analysis of physical and chemical indexes and electronic senses, the relationship between bacterial flora, organic acid, taste, and aroma in fermented grains was clarified. The results indicated that *Lactobacillus* was the main bacteria in Dacha, and the mean relative content was 97.53%. The bacteria within Ercha samples were *Pseudomonas* and *Bacillus*, mean relative content was 37.16 and 28.02%, respectively. The diversity of bacterial communities in Ercha samples was significantly greater than that in Dacha samples. The correlation between *Lactobacillus* and organic acids, especially lactic acid, led to the difference between Dacha and Ercha organic acids, which also made the *pH* value of Dacha lower and the sour taste significantly higher than Ercha. *Lactobacillus* was significantly positively correlated with a variety of aromas, which made Dacha the response value of aromas higher. In addition, *Bacillus* had a significant positive correlation with bitterness and aromatic compounds, which led to a higher response value of bitterness in Ercha and made it present an aromatic aroma. This study provides an in-depth analysis of the difference between different stages of Fen flavor Baijiu, and theoretical support for the standard production and improvement in quality of Fen flavor Baijiu in the future.

## Introduction

Chinese Baijiu has a long history of manufacture over thousands of years. Due to its unique taste, it is widely accepted by the indigenous population and an essential component of both regular and festive occasions (Gong, 1993; Hao et al., 2005). Chinese Baijiu is a clear and transparent distilled liquor with an alcohol content of between 38 and 65% (vol/vol) (Fan and Qian, 2006). It not only plays a vital role in China’s economic and social development but is also an important part of China’s food industry (Xu et al., 2010; Zheng and Han, 2016). From January to June 2019, cumulative output was 3.976 million kiloliters, a year-on-year increase of 2.2%. In addition, Chinese Baijiu, brandy, and vodka are regarded globally as among the most renowned distilled alcoholic beverages with the highest sales (Liu and Sun, 2018). Hence, it is important for enterprises that produce Chinese Baijiu to have a comprehensive understanding of its fermentation process so that the method can be improved and the quality of the Baijiu enhanced.

There are 12 representative aromas in Chinese Baijiu: Luzhou flavor, Fen flavor (Light flavor), Maotai flavor, Sanhua flavor, Mixed flavor, Feng flavor, Te flavor, Sesame flavor, Laobaigan flavor, Jiugui flavor, Dong flavor, and Chi flavor. Of these, the first three are the most common (Liu and Sun, 2018). The raw materials, starter and technological process of different flavor Baijiu are different, the principal raw materials used in the production are sorghum, rice, wheat, and other grains, and a wide variety of fermentative agents, such as Daqu, Xiaoqu, Bran Koji, *etc.* the main processes are fermentation, distillation, mixing, aging and so on (Fan and Qian, 2006; Wang et al., 2011; Zheng et al., 2011). Shihua -Baijiu is Fen flavor Baijiu.

During brewing, a considerable variety of microorganisms from diverse sources (Daqu, environment, raw material, etc.) can result in extremely complex changes to the final product (Cai et al., 2021c). These microorganisms can be affected by the surrounding air, water, soil, and other factors, and can synthesize esters, acids in addition to other metabolites that can improve the quality of the Baijiu (Wang C. L. et al., 2008; Li P. et al., 2016; Fan et al., 2018). There are many kinds of microorganisms in Fen flavor Baijiu. The bacteria are an important source of many enzymes such as protease and amylase, which produce a large amount of organic acids and affect the aroma and taste of Baijiu. Fungi are the main source of alcohol production and they also produce higher alcohols, aldehydes, and terpenoids, etc. (Zheng et al., 2012; Wang and Xu, 2015; Wu et al., 2015; Pang et al., 2018; Wang and Xu, 2019). Solid state fermentation, repeated batch fermentation, staged distillation and extraction are typical steps in the production of Fen flavor Baijiu (Liu and Sun, 2018). Fen flavor Baijiu is obtained following two fermentation and distillation cycles with raw material. Dacha fermented grains (Dacha) is produced by adding distiller’s yeast to the raw materials through initial fermentation, then Dacha-Baijiu is obtained by distilling Dacha. To fully utilize the raw materials, a new distiller’s yeast is added to the distilled Dacha, and the solid substrate after further fermentation was Ercha fermented grains (Ercha), the Ercha-Baijiu is obtained by distilling Ercha. Therefore, there may be differences in the microbes in Dacha and Ercha that affect the physical and chemical indexes and flavor of fermented grains. It may cause two batches of finished Baijiu which do not have exactly the same composition, each having its own unique flavor. However, current research on the brewing process of Fen flavor Baijiu has mostly focused on the starter, material treatment and fermentation environment, with none that have studied the fermented grains of Fen flavor Baijiu (Zheng et al., 2012; Wang and Xu, 2015; Fan et al., 2018; Pang et al., 2018, 2020).

Only a few species can be identified using traditional plate culture to study the microbial community structure. Other methods that do not rely on plate culture, such as denaturing gradient gel electrophoresis (DGGE), are expensive and the ability to detect non-cultivable microbes is also limited (Zhang et al., 2005; Zhang X. et al., 2014). By contrast, high-throughput sequencing (HTS) is highly advantageous. On one hand, it can quickly, accurately, and comprehensively detect microbes in samples. On the other hand, it can provide positioning results for analysis, and can determine the reading times of different Operational Taxonomic Units (OTUs) in the template, increasing the sequencing depth of microbial populations, reducing experimental costs (Manichanh et al., 2008; Mardis, 2008; Loman et al., 2012; Cocolin et al., 2013; Kozich et al., 2013). To date, HTS has been used to analyze microbial communities in the fields of environment, organisms, and food, etc. (Roh et al., 2010; Ye et al., 2011; Calasso et al., 2016; Johnston et al., 2017; Yong et al., 2017). The maximum read length of HTS cannot fully cover the full range of 16S ribosomal Ribonucleic Acid (16S rRNA) gene (Nelson et al., 2014). Amplification and sequencing of the V3-V4 region of the macrogenomic 16S rRNA gene have been widely used to study bacterial communities in food (Polka et al., 2015; Li and Yue, 2016; Sun et al., 2018). Electronic tongue and electronic nose can be used to simulate human taste and smell, respectively. They can quickly relay intuitive data and accurately evaluate food taste and aroma (Vlasov et al., 2005; Graham et al., 2010). These two technologies have been applied in the food industry, such as food storage, identification, quality monitoring, processing technology optimization, and so on (Gómez et al., 2006; Rodriguez-Mendez et al., 2014; Peris and Escuder-Gilabert, 2016; Wijaya et al., 2017; Cai et al., 2019).

Therefore, in the present study, after extracting the metagenomic Deoxyribonucleic Acid (DNA) from the fermented grain samples, the V3-V4 target region of 16S rRNA gene was amplified and sequenced to confirm the bacterial diversity, and multi-faceted statistical analysis of the bacteria within Dacha and Ercha. Then the taste and aroma of fermented grains were detected by electronic sensory and high performance liquid chromatography. The correlation between bacteria in fermented grains and various indexes of fermented grains was analyzed, and the differences between Dacha and Ercha were compared. In order to analyze the causes of the difference between Dacha-Baijiu and Ercha-Baijiu in Fen flavor Baijiu from microorganism perspective, it provides important theoretical support for stabilizing the quality of Fen flavor Baijiu and improving its technology in production.

## Materials and Methods

### Sample Treatment

The samples were collected from Shihua Winery Co., Ltd., Hubei. In 10 different fermentation jars, 10 Dacha samples after the first fermentation and 10 Ercha samples after the second fermentation were collected. The samples were collected by the five point sampling method, and fermented grains in the upper, middle, and lower layers of each fermentation jar were collected and mixed (500 g). Then fermented grains samples of different jars were put into a separate sterile bag for subsequent analysis within 24 h.

### Extraction of Metagenomic DNA of Microbes in Fermented Grains

QIAGEN DNeasy mericon Food Kit (QIAamp DNA microbiome kit, QIAGEN Inc.) was used to extract metagenomic DNA of 2 g samples. Micro-ultraviolet spectrophotometer and 1% agarose gel electrophoresis was used to detect DNA purity and concentration to obtain high quality and concentration of microbial metagenomic DNA (Cai et al., 2021a).

### Polymerase Chain Reaction Amplification of Bacterial DNA

Polymerase chain reaction (PCR) was used to amplify the V3-V4 region of the bacterial metagenomic 16S rRNA gene, and the primers used were forward primer 338F (5′-ACTCCTACGGGAGGCAGCAG-3′) and reverse primer 806R (5′-GGACTACHVGGGTWTCTAAT-3′). The extracted DNA was used as a template for 16S rRNA gene PCR amplification, and barcoded primers were used for PCR. The following amplification system was used: 4 μL TransStart™ 5 × FastPfu buffer, 2 μL 2.5 mM dNTPs mix, 0.5 μL 5 μM 338F and 806R, 0.4 μL TransStart™ FastPfu polymerase, 10 ng DNA template, and 12.6 μL ultrapure water. The amplification reaction conditions were: pre-denaturation at 95°C for 3 mins, denaturation at 95°C for 30 s, annealing at 55°C for 30 s, extension at 72°C for 45 s, the cycle repeated 30 times, and finally full extension at 72°C for 10 min (Zhao et al., 2020). The amplified PCR products were analyzed using a 1% agarose gel, and an AxyPrep DNA gel recovery kit was used to recover the qualified products for sequencing on an Illumina MiSeq PE300.

### Sequence Splicing and Quality Control

Firstly, double-end sequence data obtained from the MiSeq PE300 was spliced according to the overlap of the sequences. During the splicing process, the number of bases in overlapping regions was required to be ≥10 bp or with a maximum mismatch ratio ≤0.2. In addition, no barcode base mismatches were tolerated on the spliced sequences, while the number of base mismatches in the primers was required to be ≤2 bp. Finally, off-machine sequences were grouped by sample barcode information. After correction of the sequence direction, the barcodes and primers were removed, and the length of the final sequence was checked to be ≥50 bp. Any sequence not meeting the requirements above was removed, and only high-quality sequences were retained for subsequent experiments.

### Conventional Analysis

The soluble solids of the samples were determined by the automatic refractometer, and the *pH* value of the samples was determined the *pH* meter.

### Organic Acid Analysis

Analysis of organic acids was performed as follows (Cai et al., 2021b): LC-20ADXR high performance liquid chromatography (equipped with sil-20axr automatic sampler, LC-20ADXR four element low pressure gradient pump, CTO-10AS vp column incubator, SPD-M20A diode array UV visible detector), Inerttsil ODS-SP C18 column (150 mm × 4.6 mm, 5 μm) (Shimadzu, Japan). For preparing mother liquor, 0.15 g oxalic acid dihydrate, 0.30 g L-malic acid, 0.60 g citric acid monohydrate, 0.75 g tartaric acid, and 1.50 g lactic acid, acetic acid and succinic acid were weighted and constant volume to 50 ml with ultrapure water. Then took 0.1, 0.2, 0.5, 1.0, 1.4 ml of each mother liquor, added 0.2 ml 1.0 mol/L phosphoric acid, diluted to 10 ml with ultrapure water, and passed through 0.22 μm microporous membrane as standard solution. The mobile phase was 0.05 mol/L potassium dihydrogen phosphate (pH 2.7). For the analysis, 1 g of sample was mixed with 5 ml of absolute ethanol and evaporated to dryness, mixed with 5 ml of mobile phase and kept for 30 min, then centrifuged and the supernatant was taken to through 0.22 μm microporous membrane. The flow rate was 0.8 ml/min, the column temperature was 25°C, and the detection wavelength was 215 nm.

### Electronic Senses Analysis

SA 402B electronic tongue (Insent, Japan) is equipped with CA0, C00, AE1, CT0, and AAE sensors, which represent sourness, bitterness, astringency, saltiness, and umami taste, respectively (Cai et al., 2020). The C00, AE1, and AAE sensors were washed and then soaked in the reference solution for 30 s to obtain aftertaste A (astringency aftertaste), aftertaste B (bitterness aftertaste), and richness (umami aftertaste). For the analysis, 20 g sample was weighed and constant volume to 100 ml with deionized water, evenly stirred and soaked for 30 min, then centrifuged and the supernatant was collected. The sensors were immersed in the reference solution and the sample was measured for 30 s to measure the potential value of the solution, then the difference was calculated between the potential value of the reference solution and the sample to be measured to get the response value of the sample.

The sensor array in PEN 3 electronic nose (Airsense, Germany) is composed of 10 metal oxide semiconductor (MOS) chemical sensors, which have different sensing properties for different types of odorants, W1C sensor is sensitive to aromatic compounds, W5S sensor is sensitive to nitrogen oxides, W3C sensor is sensitive to ammonia and aromatic compounds, W6S sensor is sensitive to hydrides, W5C sensor is sensitive to alkanes and aromatic compounds, W1S sensor is sensitive to methyl, W1W sensor is sensitive to terpenes and sulfur-containing organic compounds, W2S sensor is sensitive to alcohols and aromatic compounds, W2W sensor is sensitive to aromatic compounds and sulfur-containing organic compounds and W3S sensor is sensitive to long-chain alkanes (Gómez et al., 2006; Cai et al., 2020). For the analysis, 10 g sample was weighed and placed in an airtight vial for 30 min, the cleaning time of the instrument was 95 s and the measuring time was 60 s. The ratio of the resistance of the aroma in samples to the resistance of the air was the response value.

### Statistical and Bioinformatics Analysis

Quantitative Insights into Microbial Ecology software (QIIME, v 1.7.0) was used for bioinformatics analysis, such as identification of species and relative content (Caporaso et al., 2010b). The PyNAST tool was used to calibrate and align sequences (Caporaso et al., 2010a). The UCLUST algorithm was used for two-step sequence division, dividing sequences with 100% similarity, then further dividing sequences with 97% similarity, thereby establishing OTUs (Edgar, 2010). The ChimeraSlayer algorithm was used to detect and remove OTUs containing chimeric sequences (Haas et al., 2011). A representative sequence was then selected from each remaining OTU, and the Ribosomal Database Project (RDP) tool and Greengenes database were used to compare sequence homology of each OTU at the level of phylum, class, order, family, and genus, thereby identifying their taxonomic status and clarifying the relevant information of the bacteria within fermented grains (DeSantis et al., 2006; Cole et al., 2007). The abundance and diversity of the microbial community were ascertained from the Observed Species index and Simpson index in terms of alpha diversity (Cai et al., 2021b). Principal coordinate analysis (PCoA) was conducted using unweighted and weighted UniFrac distances. A weighted pair group method with arithmetic mean analysis (WPGMA) and an unweighted pair-group method with arithmetic mean analysis (UPGMA) were used to perform clustering, from which beta diversity of bacterial community structure in the different samples was determined. Mann Whitney *U* test was used to determine the significance of Dacha and Ercha data. The sequence had been uploaded to the NCBI database, and the number is “PRJNA772945.”

### Plotting and Data Processing

R-3.6.0 application, MATLAB 2016b, and Origin 2017 software were used to plot all graphs. SAS V8 and Past software were used for correlation and difference analysis, Past software was used for ANOVA, Origin 2017 software was used for PCA.

## Results and Discussion

### Physical and Chemical Indexes

**FIGURE 1:**
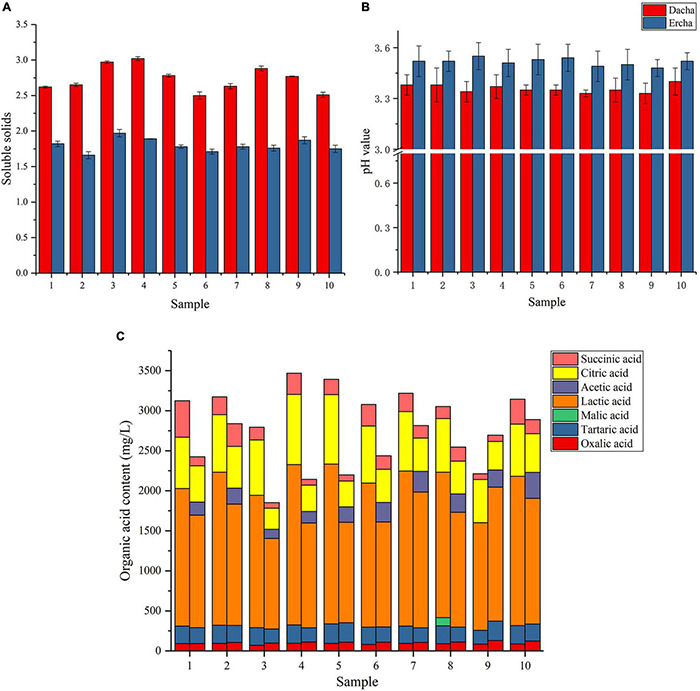
Soluble solid content **(A)**, *pH* value **(B)**, and organic acid content **(C)** of fermented grains.

The soluble solids, *pH*, and seven organic acids in fermented grains were measured and detected. It can be seen that the soluble solid content in Dacha samples was greater than Ercha, and there was a significant difference between the two groups of samples (*P* < 0.001) (Figure 1A). Soluble solids include nutrients such as sugars, acids, vitamins, and so on, the decrease of soluble solids showed that the nutrients in fermented grains could be more fully utilized through secondary fermentation (Kleinhenz and Bumgarner, 2012). It could be seen that the *pH* values of Dacha samples were lower than Ercha samples, and there was a significant difference between the two groups of samples (Figure 1B). In Figure 1C, the average total content of organic acids in Dacha was greater than Ercha, and Dacha contained a large amount of lactic acid The average content of citric acid, tartaric acid, lactic acid, and succinic acid in the Dacha sample was greater than Ercha, and the content of oxalic acid was less than Ercha. A large amount of acetic acid was detected in Ercha, but not in Dacha.

### Basic Sequencing

Of the 20 Fen flavor fermenting grains samples collected in the present study, a total of 1,104,966 original 16S rRNA gene sequences were produced. After quality control processing, 1,087,274 high-quality sequences remained in each sample producing a mean of 54,364 sequences.

Sequences that matched with more than 97% similarity were classified as an OTU. 6,944 OTUs remained after removing chimera, with a mean value of 1,599 in each fermented grain sample. Using OTU classification, the sequences were classified into 24 phyla, 65 classes, 112 orders, 195 families, and 371 genera. Only 0.05 and 1.47% of the sequences were not identified at the phylum or genus level, respectively. The composition of Dacha was identified as 15 phyla, 30 classes, 46 orders, 135 families, and 145 genera, while Ercha were identified as 24 phyla, 61 classes, 109 orders, 194 families, and 367 genera (Table 1).

**TABLE 1:** Sequencing information and diversity index of bacteria in samples.

Sample	Sequences	OTUs	Phylum	Class	Order	Family	Genus	Observed Species	Simpson
D01	51432	795	5	9	17	21	23	1309	0.57
D02	50507	919	5	11	19	22	20	1573	0.66
D03	52714	768	3	4	9	12	13	1302	0.57
D04	52768	800	6	8	13	16	18	1319	0.60
D05	51902	709	6	9	14	17	18	1285	0.57
D06	54862	707	5	7	11	13	13	1196	0.55
D07	50408	858	11	24	38	39	37	1484	0.60
D08	53572	874	7	14	20	26	28	1427	0.58
D09	54968	872	6	9	15	21	19	1409	0.59
D10	52908	768	7	9	13	13	15	1234	0.55
E01	57208	1084	11	29	59	95	153	1638	0.91
E02	57590	1073	16	33	56	77	101	1567	0.87
E03	59992	1122	17	38	68	89	112	1648	0.93
E04	54103	1034	13	28	55	90	121	1626	0.86
E05	61956	1526	24	48	75	114	163	2001	0.96
E06	63820	1122	13	32	59	85	112	1694	0.88
E07	51775	850	8	19	33	52	63	1272	0.87
E08	51677	890	18	39	65	98	115	1395	0.83
E09	44548	911	19	40	59	89	125	1537	0.86
E10	58564	780	9	16	28	36	38	1239	0.79

*D: Dacha. E: Ercha.*

### Alpha Diversity

**FIGURE 2:**
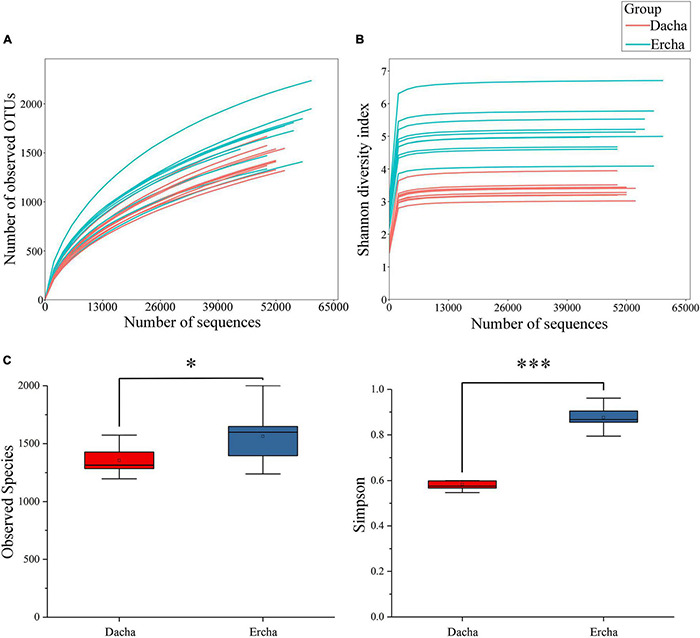
Dilution **(A)** and Shannon index **(B)** curves, and differences in alpha diversity **(C)** (NS: *P* > 0.05. *0.01 < *P* < 0.05, ^***^P < 0.001).

The Dilution curve displayed a gradual upward trend with the number of sequences increasing (Figure 2A). Each Shannon index curve exhibited a plateau stage where the number of sequences was approximately 10,000 (Figure 2B). These reflect that the vast majority of bacterial diversity were captured, although new species could still be discovered by expanding the coverage. Moreover, the Dilution and the Shannon index curves were used to further evaluate the sequencing depth to determine whether it met the requirements of subsequent bioinformatics analysis (Cai et al., 2021a). Therefore, that new species might have been detected if the number of sequences is higher, but the diversity of bacteria would not be significantly greater, and the 1,104,966 sequences obtained in the present study met the technical requirements of the analysis.

Where the sequencing depth was 44,010, the alpha diversity of Dacha and Ercha samples were further analyzed. The mean values of Observed Species and Simpson indexes of Dacha were 2255.87 and 0.58, while those of Ercha were 2395.81 and 0.88 (Table 1). The mean values of all indexes in Ercha were higher than those in Dacha.

Using difference analysis, there was a statistical difference in the Observed Species index between Dacha and Ercha, and a significant difference in the Simpson index (Figure 2C). In alpha diversity, and Observed Species index can estimate the number of species and OTUs in the sample, reflecting species richness. and Simpson index can simultaneously reflect the abundance and uniformity of species in the sample, thereby reflecting the diversity of species (Simpson, 1949). Thereby, that the difference in diversity of bacteria between Dacha and Ercha was significant, and that the diversity and the abundance of bacteria in Ercha were greater than that in Dacha.

### Analysis of Phyla and Genera of Bacteria

**FIGURE 3:**
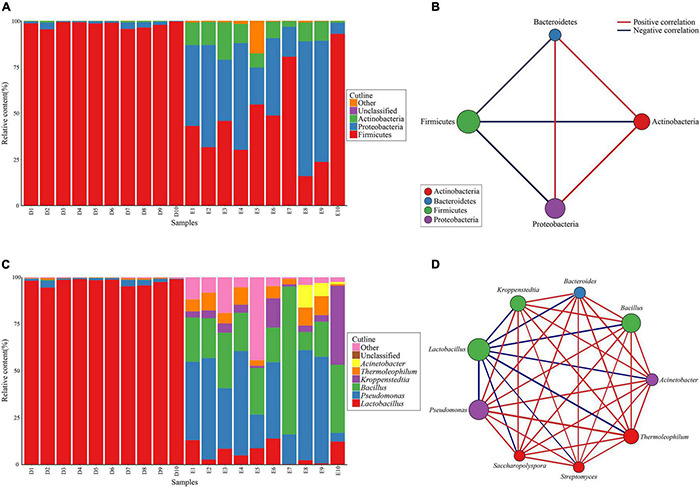
Composition and correlation of bacterial phyla **(A,B)** and genera **(C,D)** with relative content more than 1%.

For all Dacha and Ercha samples, the bacterial phyla, whose mean cumulative relative content was greater than 1% were counted, belonged mostly to Firmicutes, Proteobacteria, and Actinobacteria. Although the mean cumulative relative content of Bacteroidetes was greater than 1%, they were mostly present in Ercha samples, with a mean cumulative relative content of 2.15%, and less than 0.01% in Dacha samples, therefore not plotted in Figure 3A. Only 0.05% of the bacteria could not be identified at the phylum level. In Dacha samples, the relative Firmicutes content was as high as 97.97%, while the other phyla accounted for only 2.01%. In Ercha samples, the relative proportion of bacteria that were Firmicutes was 45.57%, Proteobacteria 40.27%, and Actinobacteria 9.72% (Figure 3A).

Correlation analysis was performed on Firmicutes, Proteobacteria, Actinobacteria, and Bacteroidetes in Dacha and Ercha. For the 20 Dacha and Ercha samples, the mean cumulative relative content of Firmicutes was 72.77%, Proteobacteria was 20.94%, Actinobacteria was 5.06% and Bacteroidetes was 1.08%. Proteobacteria, Actinobacteria, and Bacteroidetes were positively correlated with each other, while Firmicutes were negatively correlated with the other three phyla (Figure 3B).

All bacterial genera that had a mean cumulative relative content higher than 1% in Dacha and Ercha samples were counted. Principally they were *Lactobacillus*, *Pseudomonas*, *Bacillus*, *Kroppenstedtia*, *Thermoleophilum*, and *Acinetobacter*. Only a mean value of 0.73% of the bacteria could not be identified at the genus level. In Dacha samples, the mean cumulative relative content of *Lactobacillus* was 97.53%, and *Pseudomonas* was 1.56%. In Ercha samples, the mean cumulative relative content of *Pseudomonas* was 37.16%, *Bacillus* was 28.02%, *Kroppenstedtia* was 8.70%, *Lactobacillus* was 7.15%, *Thermoleophilum* was 6.24%, and *Acinetobacter* was 1.82% (Figure 3C). The mean cumulative relative content of bacteria belonging to *Bacteroides*, *Streptomyces*, and *Saccharopolyspora* in Ercha samples was higher than 1% but very low in Dacha samples, and less than 1% in Dacha and Ercha, not shown in Figure 3C.

Correlation analysis was performed on genera whose content was greater than 1% in Dacha and Ercha. For *Thermoleophilum*, *Streptomyces* and *Saccharopolyspora* that belong to Actinobacteria, *Bacteroides* belonging to Bacteroidetes, *Bacillus* and *Kropsepenstedtia* belonging to Firmicutes and *Pacterobacteria* belonging to Proteinobacteria, the mean cumulative relative content was positively correlated with each other, while *Lactobacillus* belonging to Firmicutes was negatively correlated with the other 7 genera (Figure 3D).

Firmicutes and *Lactobacillus* were the most abundant bacterial phylum and genera of Dacha. These results were similar to the previous research on microorganisms in Fen flavor Baijiu, and it might be related to the Daqu added in the brewing process of Baijiu (Zhang X. et al., 2014; Wang and Xu, 2015; Fan et al., 2018). Previous studies have also found that lactic acid bacteria exist widely in Baijiu production environment, along with distiller yeast and fermented grains (Wang H. Y. et al., 2008; Wang and Guo, 2011; Wang et al., 2018). Almost all the bacteria in Dacha were *Lactobacillus* of Firmicutes. Lactic acid bacteria can produce a variety of substances with antibacterial activity, so it can inhibit the growth of other bacteria (Piard and Desmazeaud, 1992; Oyewole, 1997). This shows that it is the bacteria that play a major role in the first fermentation process and inhibit the growth of other bacteria, which also explains why Firmicutes and *Lactobacillus* are negatively correlated with other phyla and genera. Sorghum, the main raw material in the first fermentation process in the production of Fen flavor Baijiu is rich in nutrients and provides a good environment for bacterial growth (Zhang X. et al., 2014; Xiong et al., 2019). *Lactobacillus* grow principally in a nutrient-rich environment and they require carbohydrates, amino acids, vitamins, and other nutrients to satisfy their growth (Tannock, 2004; Gong et al., 2020). Previous studies have shown that the pretreatment of raw materials introduces microorganisms such as lactic acid bacteria from water, floor, and air, which has become an important source of lactic acid bacteria other than distiller’s yeast (Jin et al., 2017; Wang et al., 2018; Pang et al., 2020). Therefore, there is much *Lactobacillus* in Dacha after the first fermentation. However, the second fermentation is an additional fermentation using distilled Dacha as fermentation substrate. After the first fermentation, the nutritional components of the raw materials changed greatly, and the temperature of the distillation step is about 80°C, which kills most bacteria such as *Lactobacillus* in Dacha, but excluding bacteria such as *Bacillus* that can withstand higher temperature (Movahedi and Waites, 2002; Breidt and Costilow, 2004; Coleman et al., 2007). In addition, besides *Lactobacillus*, distiller’s yeast also contains a large number of bacteria, such as *Bacillus*, *Thermophilum* and so on. New distiller’s yeast was added in second fermentation, and the raw material pretreatment step that can obtain a large amount of *Lactobacillus* is no longer carried out (Zhang L. et al., 2014; Zheng et al., 2014; Xiao et al., 2017). This may lead to the lower relative content of *Lactobacillus* in Ercha than Dacha, the increase of the relative content of *Bacillus*, *Thermophilum*, etc. The decrease of *Lactobacillus* also reduces the inhibitory effect on other bacteria. Finally, the bacterial diversity in Ercha is higher than Dacha.

### Beta Diversity

**FIGURE 4:**
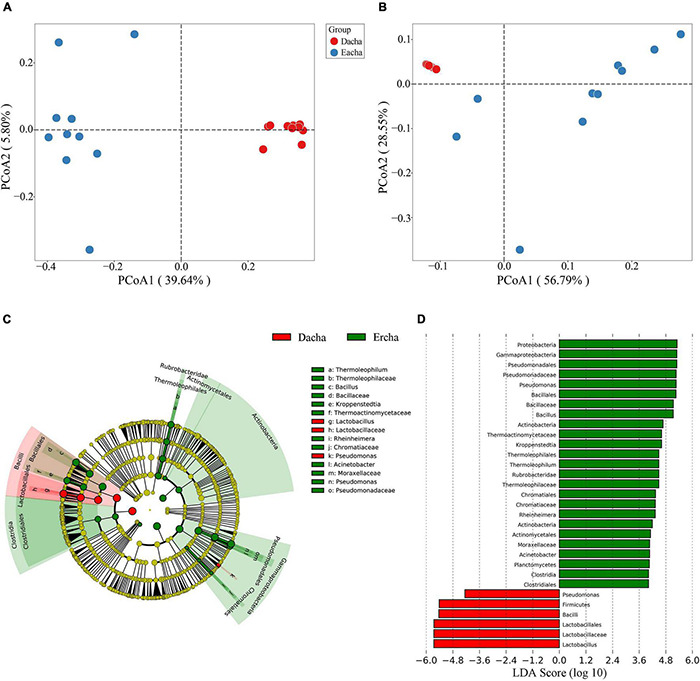
PCoA based on unweighted **(A)** and weighted **(B)** UniFrac distance, LEfSe and LDA analysis indicating the cladogram **(C)** and LDA scores **(D)**.

Principal coordinate analysis of the distance of unweighted and weighted UniFrac was used to analyze the beta diversity of samples. From the PCoA of unweighted UniFrac values, the first and the second principal components accounted for 39.64 and 5.80%, respectively. Dacha samples were strongly distributed within the first and fourth quadrants, while Ercha samples were sparsely distributed in the second and third quadrants (Figure 4A). From the PCoA of weighted UniFrac values, the contribution of the first and second principal components was 56.79 and 28.55%, respectively. Dacha samples were strongly distributed in the second quadrant, while, Ercha samples were distributed in the first, third, and fourth quadrants (Figure 4B). In beta diversity, unweighted UniFrac values only reflect changes in species, while weighted UniFrac values simultaneously reflect a change in species and the abundance of species (Lozupone and Knight, 2005; Lozupone et al., 2007). The spatial arrangement of Dacha and Ercha samples in PCoA were different, and the two sets of samples were completely separated without overlap. This showed that there were obvious differences in the species and abundance of bacteria between Dacha and Ercha.

Linear discriminant analysis Effect Size (LEfSe) based on OTUs characterized bacterial community structure in Dacha and Ercha samples. Cladogram indicated the phylogenetic distribution of bacteria between Dacha and Ercha, the green and red nodes were the most important species in Ercha and Dacha groups, respectively, and the yellow nodes represented the species with no significant difference (Figure 4C). Line Discriminant Analysis (LDA) scores showed the significant bacterial difference between Dacha and Ercha, as shown 31 bacterial clades, were determined to be significantly discriminant with an LDA threshold of 4 (Figure 4D). The abundance of Firmicutes, *Pseudomonas* in Dacha was much higher. Proteobacteria, Firmicutes, Actinobacteria, and Planctomycetes showed abundance advantage in Ercha. Dacha in genus level was characterized by a preponderance of *Lactobacillus*, and Ercha was characterized by *Pseudomonas*, *Bacillus*, *Kroppenstedtia*, and *Thermoleophilum*. The microbial biomarkers showed a significant difference in abundance between the Dacha and Ercha. Therefore, *Lactobacillus*, *Pseudomonas*, *Bacillus*, *Kroppenstedtia*, and *Thermoleophilum* were the main bacteria causing the difference in the bacterial community structure between Dacha and Ercha.

### Operational Taxonomic Unit Statistics and Analysis

Rank-Abundance curve is used to display relative species abundance and the diversity of species within samples. The overall trend of Dacha samples was consistent and the length of the horizontal axis was similar, different from the finding in Ercha samples (Supplementary Figure 1). In addition, the curves of almost all Dacha samples were steeper, and the length of the horizontal axis less, than that of Ercha samples. The rank abundance curve can visually depict both species richness and species evenness, and a steep gradient indicates low evenness and a shallow gradient indicates high evenness (Whittaker, 1965). Therefore, the abundance and evenness of the bacteria in Dacha samples were lower than those inErcha samples, and the diversity of bacteria in Dacha was lower than that in Ercha.

In the present study, a total of 6,943 OTUs were identified, of which only 23 were core OTUs, accounting for only 0.33% of the total number, but comprising 774,808 sequences, accounting for 71.26% of the total sequences. For Dacha samples, OTU20042 was in the highest relative proportion among the core OTUs with a relative abundance of 63.7%, followed by OTU23681 and OTU14635 with a relative abundance of 9.87 and 2.42%, respectively, the relative abundance of other core OTUs were all less than 1%. OTU20042, OTU23681, and OTU14635 belong to *Lactobacillus*, a member of the Firmicutes. For Ercha samples, OTU12679 displayed the highest relative content of the core OTUs accounting for 22.97%, followed by OTU18345, OTU10099, OTU10577, OTU15793, OTU20042, and OTU4512, with a relative abundance of 9.34, 8.85, 8.27, 7.73, 3.79, and 1.11%, respectively, all other core OTUs constituting less than 1% of the total. OTU12679 and OTU10099 were *Pseudomonas* from Proteobacteria, OTU18345 and OTU15793 were *Bacillus* from Firmicutes, OTU10577 was *Kroppenstedtia* from Firmicutes, OTU20042 was *Lactobacillus* from Firmicutes, and OTU4512 was *Saccharopolyspora* from Actinobacteria. For all samples, 9 core OTUs were *Lactobacillus*, namely OTU6139, OTU6440, OTU8424, OTU16597, OTU14635, OTU20042, OTU18594, OTU23681, and OTU8198, respectively, and their relative abundance was 77.35% (Table 2).

**TABLE 2:** Information on the bacterial phylum and genus of core OTUs.

OTU	Phylum	Genus	Relative content in Dacha/(%)	Relative content in Ercha/(%)	*P* value
OTU5877	Actinobacteria	*Thermoleophilum*	0.01 (0.01, 0–0.04)*	0.09 (0.09, 0.02–0.16)	0.00033
OTU23203	Actinobacteria	*Thermoleophilum*	0.01 (0.01, 0–0.03)*	0.3 (0.31, 0.02–0.73)	0.00025
OTU9419	Actinobacteria	*Thermoleophilum*	0.02 (0.01, 0–0.05)*	0.07 (0.07, 0.01–0.14)	0.00291
OTU4512	Actinobacteria	*Saccharopolyspora*	0.01 (0.01, 0–0.03)*	1.11 (0.15, 0.03–6.48)	0.00018
OTU20739	Proteobacteria	*Pseudomonas*	0.01 (0.01, 0–0.04)*	0.37 (0.34, 0.04–0.62)	0.00022
OTU18084	Proteobacteria	*Pseudomonas*	0.03 (0.02, 0–0.07)*	0.05 (0.04, 0.01–0.11)	0.16775
OTU10099	Proteobacteria	*Pseudomonas*	0.28 (0.2, 0.04–0.66)	8.85 (9.28, 1.03–13.45)	0.00018
OTU9376	Proteobacteria	*Pseudomonas*	0.05 (0.02, 0.01–0.13)	0.07 (0.06, 0.03–0.15)	0.05707
OTU12679	Proteobacteria	*Pseudomonas*	0.86 (0.6, 0.11–2.05)	22.97 (25.96, 2.74–36.88)	0.00018
OTU9151	Proteobacteria	*Pseudomonas*	0.02 (0.01, 0–0.07)*	0.07 (0.06, 0.01–0.13)	0.0067
OTU9158	Proteobacteria	*Pseudomonas*	0.03 (0.02, 0–0.07)*	0.78 (0.86, 0.08–1.27)	0.00018
OTU6139	Firmicutes	*Lactobacillus*	0.25 (0.19, 0.1–0.45)	0.01 (0.01, 0–0.05)*	0.00016
OTU6440	Firmicutes	*Lactobacillus*	0.14 (0.13, 0.1–0.29)	0.01 (0, 0–0.03)*	0.00013
OTU8424	Firmicutes	*Lactobacillus*	0.24 (0.15, 0.11–0.63)	0.01 (0.01, 0–0.02)*	0.00016
OTU16597	Firmicutes	*Lactobacillus*	0.36 (0.36, 0.3–0.4)	0.02 (0.02, 0–0.06)*	0.00017
OTU14635	Firmicutes	*Lactobacillus*	2.42 (2.43, 2.22–2.61)	0.15 (0.12, 0.01–0.38)	0.00018
OTU20042	Firmicutes	*Lactobacillus*	63.7 (64.3, 57.64–66.31)	3.79 (3.1, 0.16–9.82)	0.00018
OTU18594	Firmicutes	*Lactobacillus*	0.1 (0.1, 0.08–0.12)	0.01 (0, 0–0.04)*	0.00012
OTU23681	Firmicutes	*Lactobacillus*	9.87 (9.91, 8.32–11.36)	0.2 (0.13, 0–0.43)	0.00018
OTU8198	Firmicutes	*Lactobacillus*	0.27 (0.26, 0.21–0.36)	0.02 (0.02, 0–0.04)*	0.00017
OTU10577	Firmicutes	*Kroppenstedtia*	0.08 (0.07, 0.02–0.28)	8.27 (3.77, 1.07–41.67)	0.00018
OTU15793	Firmicutes	*Bacillus*	0.02 (0.01, 0.01–0.09)	7.73 (6.43, 2.93–21.48)	0.00015
OTU18345	Firmicutes	*Bacillus*	0.03 (0.02, 0–0.08)*	9.34 (7.66, 3.27–26.21)	0.00017

*Since the relative content of the OTU in a sample is low, it will be displayed as 0 after keeping two decimal places.

The correlation analysis on the relative abundance values of the core OTUs in Dacha and Ercha samples indicates that the relative abundance of OTU18084 and OTU9376, which were *Pseudomonas* from Proteobacteria, were not significantly different (*P* = 0.168 and 0.057), while other core OTUs displayed significant differences (*P* < 0.01). Of these, the mean relative content of OTU18084 and OTU9376 only accounted for 0.04 and 0.06%, respectively, of the core OTUs of Dacha and Ercha samples (Table 2). In addition, there were 34 OTUs that were unique from all Dacha samples, 33 of which were *Lactobacillus* from Firmicutes, and one that could not be identified at the level of the genus, present at a level of only 0.007–0.01%. There were 96 OTUs unique in Ercha samples, 88 of which were *Bacillus* from Firmicutes, one was *Oceanobacillus* from Firmicutes, one was *Staphylococcus* from Firmicutes, two were *Pseudomonas* from Proteobacteria, and four were *Thermoleophilum* from Actinobacteria, with a mean relative content of only 0.008 ∼ 0.094%.

The statistics and analysis of OTU indicate that the abundance of the bacteria in the different Dacha samples was similar. Although only a few core OTUs were not significantly different between the two sets of samples, they only accounted for a very small percentage of Dacha and Ercha samples. Hence, the main bacterial species causing the difference in bacterial diversity between Dacha and Ercha were the same, and the difference in the number of the principal bacteria was the cause of the difference in the diversity of the two sets of samples. This showed that the dominant *Lactobacillus* in Dacha samples was the main reason for the diversity difference between the two samples.

### Prediction of Bacterial Functional Phenotypes

The potential prediction for phenotypic functions of bacterial communities in Dacha and Ercha detected 9 potential microbiome phenotypes including: aerobic, anaerobic, contains mobile elements, facultatively anaerobic, forms biofilms, Gram-negative, Gram-positive, potentially pathogenic, and stress tolerant (Supplementary Figure 2). The differences in the abundance of all predicted phenotypic functions were very significant (*P* < 0.01), indicating that the bacterial communities with these functions affected secondary fermentation. It can be seen from phenotypic prediction, in the functions of facultatively anaerobic and Gram-positive, the functional abundance of microorganisms in Dacha was higher than that in Ercha, and the other functions were that the functional abundance of Ercha was higher. Through the analysis of bacteria in fermented grains, it was found that the bacteria in Dacha were mainly *Lactobacillus*. *Lactobacillus* is a genus of Gram-positive, facultative anaerobic rod-shaped bacteria, it proliferated and produced lactic acid, thereby creating an acid environment that inhibited the growth of other bacteria (Makarova et al., 2006; Zhang et al., 2016). Therefore, the facultatively anaerobic and Gram-positive functional abundance of microorganism were higher.

### Analysis of Physical and Chemical Indexes

**FIGURE 5:**
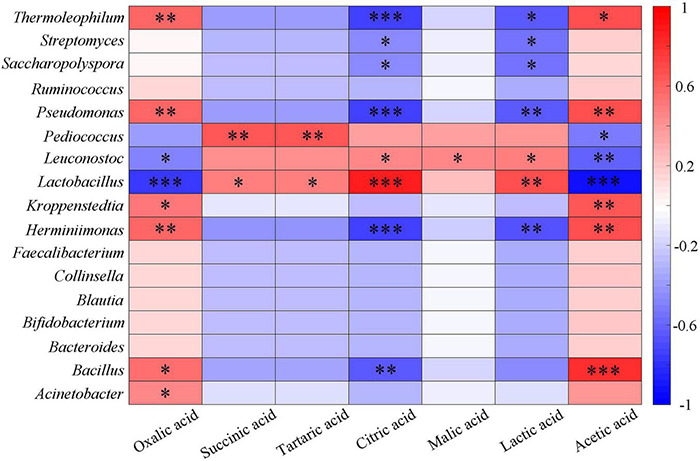
Heatmap of correlation between organic acids and major bacteria genera of fermented grains (*0.01 < *P* < 0.05, ^**^0.001 < *P* < 0.01, ^***^P < 0.001).

The existence and concentration of organic acids determine the acidity, and lactic acid bacteria hydrolyze sorghum starch to produce lactic acid that can reduce the *pH* value (Desmazeaud, 1996; Soomro et al., 2001; Blandino et al., 2003). Dacha fermented grains had higher total organic acids and contained a large amount of lactic acid, which was an important reason for the low *pH* value. The correlation between seven organic acids and the main bacterial genera was analyzed (Figure 5). It could be seen that oxalic acid had a significant positive correlation with *Herminiimonas*, *Pseudomonas*, and *Thermoleophilum* (*P* < 0.01). Succinic acid and tartaric acid, citric acid and lactic acid, malic acid and acetic acid showed a significant positive correlation with *Pediococcus*, *Lactobacillus*, *Leuconostoc*, and *Bacillus*, respectively. The correlation between other bacteria and seven organic acids showed the opposite results with *Lactobacillus*, *Leuconostoc*, and *Pediococcus*. The main end product of *Lactobacillus* fermentation is lactic acid, which plays a major role in lactic acid production. A large number of *Lactobacillus* found may be the important reason for the highest content of lactic acid in all samples (58.8%), this phenomenon was similar to previous studies on Baijiu (Zheng et al., 2012; Su et al., 2015; Li S. et al., 2016; He et al., 2019; Yang et al., 2019). More *Lactobacillus* found in Dacha, caused a significant correlation between lactic acid in the two fermented grains. In addition, acetic acid was only detected in Ercha, previous studies found that ethanol and sugar could be used by bacteria to produce acetic acid (Ljungdahl, 1986; Sengun and Karabiyikli, 2011). *Acetobacter*, *Gluconacetobacter*, *Gluconobacter*, and *Clostridium* were unique genera of bacteria in Ercha. They were some of the main bacteria producing acetic acid, and the main bacteria in Ercha, *Bacillus* had also been shown to produce acetic acid (Yan et al., 2013a; Schuchmann and Muller, 2014; Li et al., 2015). This might be the reason why a large amount of acetic acid was detected in Ercha and *Bacillus* showed a very significant positive correlation with acetic acid.

### Analysis of Taste

The eight tastes of sourness, bitterness, astringency, saltiness and umami sourness, bitterness, astringency, saltiness, umami, aftertaste A (astringency aftertaste), aftertaste B (bitterness aftertaste), and abundance (umami aftertaste) of fermented grains were detected by electronic tongue.

**FIGURE 6:**
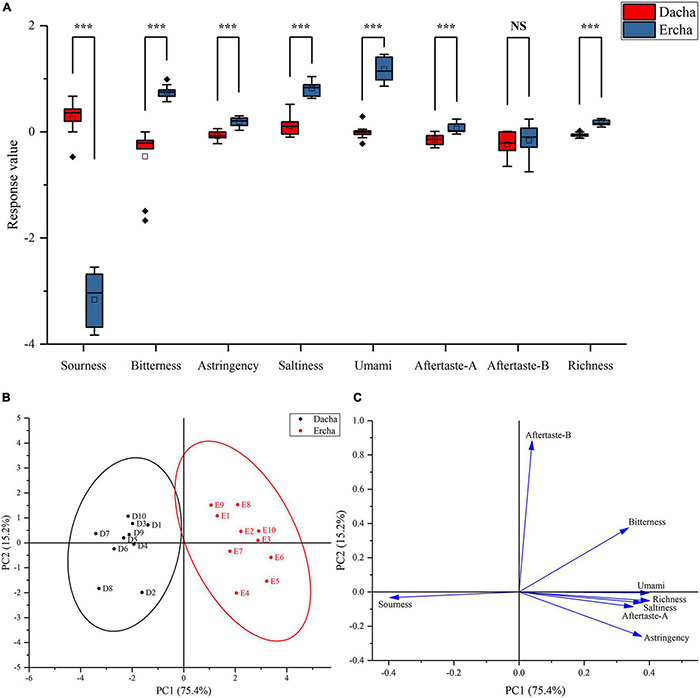
Box plot **(A)** of response value of electronic tongue, score plot **(B)**, and loading plot **(C)** of PCA (NS: *P* > 0.05, ***P < 0.001).

In addition to the bitter aftertaste, Dacha and Ercha showed extremely significant differences. Dacha samples had higher acid response values than Ercha, and other tastes had higher response values than Ercha samples. The two groups of samples had the greatest difference in response values of tastes, which were sour and bitter (Figure 6A). Through PCA, Dacha samples were distributed on the left of the Y-axis, and Ercha samples were distributed on the right of the y-axis (Figure 6B). Except that sour taste was on the left of the y-axis, other tastes were located on the right of the y-axis (Figure 6C). It showed that Dacha mainly presents sour taste, and the other tastes were mainly presented in Ercha.

**FIGURE 7:**
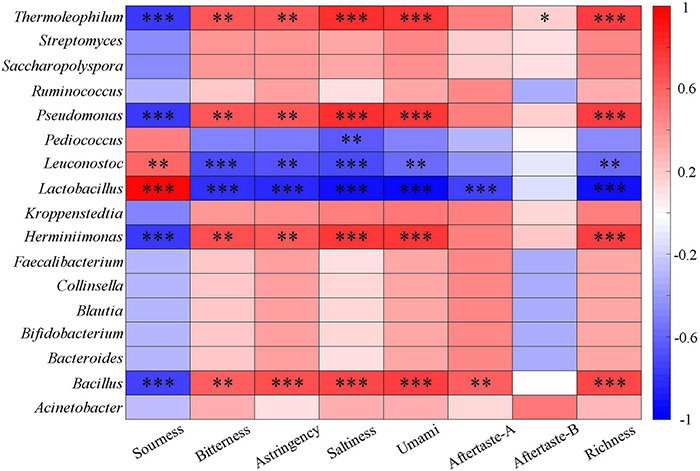
Heatmap of correlation between taste and major bacterial genera of fermented grains (*0.01 < *P* < 0.05, ^**^0.001 < *P* < 0.01, ^***^P < 0.001).

The correlation between tastes and main bacterial genera was further analyzed. *Lactobacillus* had a very significant positive correlation with a sour taste (Figure 7), which is closely related to the fact that the dominant lactic acid bacteria in Dacha produce a large amount of lactic acid during fermentation and inhibit the growth of other microorganisms (Piard and Desmazeaud, 1992; Desmazeaud, 1996). This could reduce the production of taste substances by other microorganisms, resulting in a significant negative correlation between *Lactobacillus* and other tastes. Among the tastes with significant differences, the correlation between bacteria and each taste was opposite to that of *Lactobacillus*, *Leuconostoc*, and *Pediococcus*. *Thermophilum*, *Pseudomonas*, *Hermiminonas*, and *Bacillus* were significantly positively correlated with bitter, salty, umami, and umami aftertaste, and *Bacillus* was significantly positively correlated with astringency. Astringency and bitterness are mainly produced by flavonoids and phenolic compounds. Previous studies found that *Bacillus* could produce flavonoids and phenolic compounds and improved antioxidant capacity (Boudreau, 1980; Robichaud and Noble, 1990; Dajanta et al., 2013; Kadaikunnan et al., 2015; Moayedi et al., 2016). This might be an important reason for the significant positive correlation between *Bacillus* and bitter taste, and the presence of a large number of *Bacillus* in Ercha could also lead to the higher response value of its bitter taste than Dacha.

### Analysis of Aroma

**FIGURE 8:**
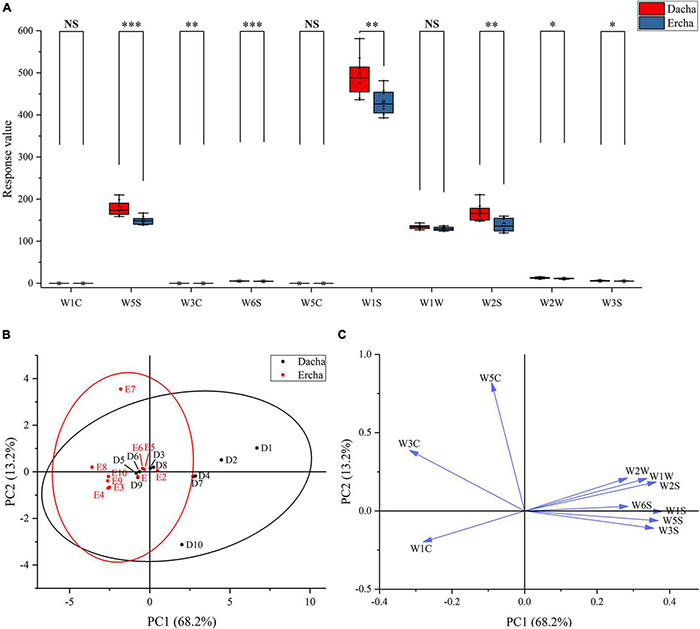
Box plot **(A)** of response value of electronic nose, score plot **(B)**, and loading plot **(C)** of PCA (NS: *P* > 0.05, *0.01 < *P* < 0.05, ^**^0.001 < *P* < 0.01, ^***^P < 0.001).

The aromas of 20 fermented grains were detected by electronic nose. The W5S and W6S response values of Dacha and Ercha showed very significant differences, W3C, W1S, and W2S had significant differences, and W1C, W5C, and W1W had no statistical differences. The response values of W1C, W3C, and W5C of Ercha were greater than those of Dacha. Dacha response values of other sensors were higher, and the range of W1S, W2S, and W5S response values were relatively largest (Figure 8A). Through PCA, Ercha was mainly distributed in the second and third quadrants. Dacha was mainly distributed in the first and fourth quadrants, W5C and W3C were distributed in the second quadrant, W1C was in the third quadrant, and other sensors were distributed in the first and fourth quadrants (Figures 8B,C). The aromas of Dacha were diverse, and the aromas of Ercha were mainly aromatic compounds.

**FIGURE 9:**
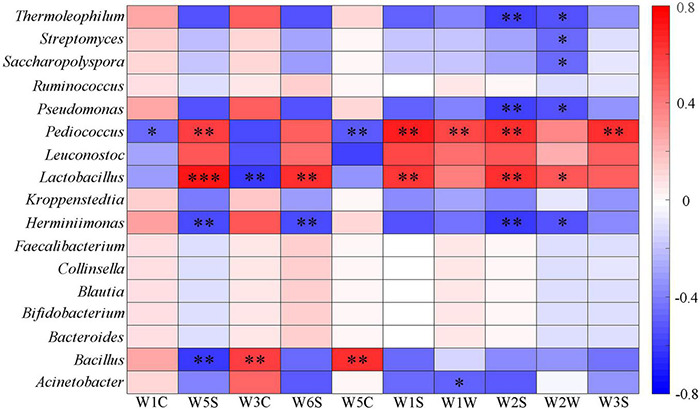
Heatmap of correlation between flavor and major bacterial genera of fermented grains (*0.01 < *P* < 0.05, ^**^0.001 < *P* < 0.01, ^***^P < 0.001).

The correlation between aromas and main bacterial genera was analyzed. It was found that the bacterial genera that mainly affected each aroma was *Acinetobacter*, *Bacillus*, *Herminiimona*, *Lactobacillus*, *Pediococcus*, *Pseudomonas*, *Saccharopolyspora*, *Streptomyces*, and *Thermoleophilum*. Among the detectors with different response values, *Lactobacillus* had a very significant positive correlation with W5S, W6S, W1S, and W2S, and *Pediococcus* had a significant positive correlation with W5S, W1S, W2S, and W3S. *Bacillus* had a significant positive correlation with W3C and W5C and a significant negative correlation with W5S. *Lactobacillus*, *Leuconostoc*, and *Pediococcus* had the opposite correlation with other bacteria on all sensors (Figure 9). *Lactobacillus* and *Pediococcus* were the dominant bacteria in the fermentation of Fen flavor Baijiu, which is positively correlated with most aroma substances. In addition, *Lactobacillus* has a positive correlation with other aromas, such as acids, esters, phenols, alcohols, and plays a key role in the formation of volatile compounds (Wang et al., 2014; Wang et al., 2016; Song et al., 2017; Pang et al., 2018). This explained why *Lactobacillus* and *Pediococcus* were significantly positively correlated with multiple aromas, and a large number of *Lactobacillus* in Dacha could be an important reason for the diversity of aromas. Previous studies had found that *Bacillus* could produce many aroma compounds, which were positively correlated with some esters and related to the metabolism of nitrogen oxides (Hoshino and Morimoto, 2008; Zheng et al., 2012; Yan et al., 2013b; Li et al., 2014; Guo et al., 2017). This might be the reason why *Bacillus* has a significant positive correlation with aromatic compounds and a negative correlation with nitrogen oxides. In addition, a large number of *Bacillus* could be the reason why Ercha mainly presents aromatic odor. In general, except W1C, W5C, and W1W, the response values detected by Dacha in each aroma were significantly higher than Ercha, which may be related to the main *Lactobacillus* in Dacha. Previous studies found that ethyl acetate was the main aroma substance in Fen flavor Baijiu, and *Lactobacillus* as a dominant genus was positively correlated with esters; its metabolites provided a basis for the composition of aromatic compounds (Su et al., 2015; Liu and Sun, 2018; Pang et al., 2018; Pang et al., 2020). This might increase the response value of aroma compounds of Dacha and contribute to the aroma formation of Baijiu.

## Conclusion

For the first time, this study investigated the bacterial populations in Dacha and Ercha fermented grains of Fen flavor Baijiu and analyzed the bacterial diversity in it. Through the detection of physical and chemical indexes and electronic senses, the relationship between bacterial microbiota and organic acid, aroma, and taste in fermented grains was clarified. The results showed that the bacterial diversity of Ercha was significantly higher than that of Dacha, *Lactobacillus* was mainly in Dacha, and *Pseudomonas* and *Bacillus* were mainly in Ercha. In addition, the two fermented grains showed great differences in physical and chemical indexes, taste, and aroma. This was due to the great difference of bacterial communities in the two fermented grains, and the bacteria that played a major role were *Lactobacillus* and *Bacillus*. This may provide some clues for in-depth analysis of the difference between different stages of Fen flavor Baijiu, and provide theoretical support for the standard production and improvement in quality of Fen flavor Baijiu in the future.

## Data Availability Statement

The original contributions presented in the study are included in the article/Supplementary Material, further inquiries can be directed to the corresponding authors.

## Author Contributions

ZG, CS, and FT designed the study. WC and ZG collected samples. XZ and YX processed samples. YX, WC, and WS analyzed the data. YX wrote the manuscript. All authors contributed to the article and approved the submitted version.

## Conflict of Interest

The authors declare that the research was conducted in the absence of any commercial or financial relationships that could be construed as a potential conflict of interest.

## Publisher’s Note

All claims expressed in this article are solely those of the authors and do not necessarily represent those of their affiliated organizations, or those of the publisher, the editors and the reviewers. Any product that may be evaluated in this article, or claim that may be made by its manufacturer, is not guaranteed or endorsed by the publisher.

## Supplementary Material

Supplementary Figure 1 Rank-abundance curve.

Supplementary Figure 2 Comparative analysis of bacterial phenotypic results.

## References

[B1] Blandino A. Al-Aseeri M. E. Pandiella S. S. Cantero D. Webb C. (2003). Cereal-based fermented foods and beverages. *Food Res. Int.* 36 527–543. 10.1016/s0963-9969(03)00009-7

[B2] Boudreau J. C. (1980). Taste and the taste of foods. *Naturwissenschaften* 67 14–20. 10.1007/bf00424498

[B3] Breidt F. Costilow R. N. (2004). *Processing and Safety.* St. Charles, IL: Pickle Packers International, Inc.

[B4] Cai W. Tang F. Zhao X. Guo Z. Zhang Z. Dong Y. (2019). Different lactic acid bacteria strains affecting the flavor profile of fermented jujube juice. *J. Food Process. Preserv.* 43:112772. 10.1111/jfpp.14095

[B5] Cai W. Wang Y. Ni H. Liu Z. Liu J. Zhong J. A. (2021c). Diversity of microbiota, microbial functions, and flavor in different types of low-temperature Daqu. *Food Res. Int.* 150:110734. 10.1016/j.foodres.2021.110734 34865753

[B6] Cai W. Tang F. Wang Y. Zhang Z. Xue Y. Zhao X. (2021a). Bacterial diversity and flavor profile of Zha-Chili, a traditional fermented food in China. *Food Res. Int.* 141:110112. 10.1016/j.foodres.2021.110112 33641979

[B7] Cai W. Wang Y. Hou Q. Zhang Z. Tang F. Shan C. (2021b). Rice varieties affect bacterial diversity, flavor, and metabolites of zha-chili. *Food Res. Int.* 147:110556. 10.1016/j.foodres.2021.110556 34399533

[B8] Cai W. C. Tang F. X. Guo Z. Guo X. Zhang Q. Zhao X. X. (2020). Effects of pretreatment methods and leaching methods on jujube wine quality detected by electronic senses and HS-SPME–GC–MS. *Food Chem.* 330:127330. 10.1016/j.foodchem.2020.127330 32569941

[B9] Calasso M. Ercolini D. Mancini L. Stellato G. Minervini F. Di Cagno R. (2016). Relationships among house, rind and core microbiotas during manufacture of traditional Italian cheeses at the same dairy plant. *Food Microbiol.* 54 115–126. 10.1016/j.fm.2015.10.008

[B10] Caporaso J. G. Kuczynski J. Stombaugh J. Bittinger K. Bushman F. D. Costello E. K. (2010b). QIIME allows analysis of high-throughput community sequencing data. *Nat. Methods* 7 335–336. 10.1038/nmeth.f.303 20383131 PMC3156573

[B11] Caporaso J. G. Bittinger K. Bushman F. D. DeSantis T. Z. Andersen G. L. Knight R. (2010a). PyNAST: a flexible tool for aligning sequences to a template alignment. *Bioinformatics* 26 266–267. 10.1093/bioinformatics/btp636 19914921 PMC2804299

[B12] Cocolin L. Alessandria V. Dolci P. Gorra R. Rantsiou K. (2013). Culture independent methods to assess the diversity and dynamics of microbiota during food fermentation. *Int. J. Food Microbiol.* 167 29–43. 10.1016/j.ijfoodmicro.2013.05.008 23791362

[B13] Cole J. R. Chai B. Farris R. J. Wang Q. Kulam-Syed-Mohideen A. S. McGarrell D. M. (2007). The ribosomal database project (RDP-II): introducing myRDP space and quality controlled public data. *Nucleic Acids Res.* 35 D169–D172. 10.1093/nar/gkl889 17090583 PMC1669760

[B14] Coleman W. H. Chen D. Li Y. Q. Cowan A. E. Setlow P. (2007). How moist heat kills spores of Bacillus subtilis. *J. Bacteriol.* 189 8458–8466. 10.1128/JB.01242-07 17890306 PMC2168948

[B15] Dajanta K. Janpum P. Leksing W. (2013). Antioxidant capacities, total phenolics and flavonoids in black and yellow soybeans fermented by *Bacillus subtilis*: a comparative study of Thai fermented soybeans (thua nao). *Int. Food Res. J.* 20 3125–3132.

[B16] DeSantis T. Z. Hugenholtz P. Larsen N. Rojas M. Brodie E. L. Keller K. (2006). Greengenes, a Chimera-Checked 16S rRNA gene database and workbench compatible with ARB. *Appl. Environ. Microbiol.* 72 5069–5072. 10.1128/aem.03006-05 16820507 PMC1489311

[B17] Desmazeaud M. (1996). Les bactéries lactiques dans l’alimentation humaine: utilisation et innocuité. *Cahiers Agric* 5 331–343.

[B18] Edgar R. C. (2010). Search and clustering orders of magnitude faster than BLAST. *Bioinformatics* 26 2460–2461. 10.1093/bioinformatics/btq461 20709691

[B19] Fan G. Sun B. Fu Z. Xia Y. Huang M. Xu C. (2018). Analysis of physicochemical indices, volatile flavor components, and microbial community of a light-flavor daqu. *J. Am. Soc. Brew. Chem.* 76 209–218. 10.1080/03610470.2018.1424402

[B20] Fan W. Qian M. C. (2006). Identification of aroma compounds in Chinese ‘Yanghe Daqu’ liquor by normal phase chromatography fractionation followed by gas chromatography[sol]olfactometry. *Flavour Fragr. J.* 21 333–342. 10.1002/ffj.1621

[B21] Gómez A. H. Hu G. Wang J. Pereira A. G. (2006). Evaluation of tomato maturity by electronic nose. *Comput. Electron. Agric.* 54 44–52. 10.1016/j.compag.2006.07.002

[B22] Gong M. Zhou Z. Jin J. Yu Y. Liu S. Han X. (2020). Effects of soaking on physicochemical properties of four kinds of rice used in Huangjiu brewing. *J. Cereal Sci.* 91:102855. 10.1016/j.jcs.2019.102855

[B23] Gong W. (1993). A historical survey of chinese wine culture. *J. Pop. Cult.* 27 57–74. 10.1111/j.0022-3840.1993.00057.x

[B24] Graham L. J. Haddad R. Medhanie A. Roth Y. Harel D. Sobel N. (2010). Predicting odor pleasantness with an electronic nose. *PLoS Comput. Biol.* 6:e1000740. 10.1371/journal.pcbi.1000740 20418961 PMC2855315

[B25] Guo C.-J. Chang F.-Y. Wyche T. P. Backus K. M. Acker T. M. Funabashi M. (2017). Discovery of reactive microbiota-derived metabolites that inhibit host proteases. *Cell* 168 517.e–526.e. 10.1016/j.cell.2016.12.021 28111075 PMC5302092

[B26] Haas B. J. Gevers D. Earl A. M. Feldgarden M. Ward D. V. Giannoukos G. (2011). Chimeric 16S rRNA sequence formation and detection in Sanger and 454-pyrosequenced PCR amplicons. *Genome Res.* 21 494–504. 10.1101/gr.112730.110 21212162 PMC3044863

[B27] Hao W. Chen H. Su Z. (2005). China: alcohol today. *Addiction* 100 737–741. 10.1111/j.1360-0443.2005.01036.x 15918802

[B28] He G. Huang J. Zhou R. Wu C. Jin Y. (2019). Effect of fortified daqu on the microbial community and flavor in Chinese strong-flavor liquor brewing process. *Front. Microbiol.* 10:56. 10.3389/fmicb.2019.00056 30761106 PMC6361764

[B29] Hoshino Y. T. Morimoto S. (2008). Comparison of 18S rDNA primers for estimating fungal diversity in agricultural soils using polymerase chain reaction-denaturing gradient gel electrophoresis. *Soil Sci. Plant Nutr.* 54 701–710. 10.1111/j.1747-0765.2008.00289.x

[B30] Jin G. Zhu Y. Xu Y. (2017). Mystery behind Chinese liquor fermentation. *Trends Food Sci. Technol.* 63 18–28. 10.1016/j.tifs.2017.02.016

[B31] Johnston D. Earley B. Cormican P. Murray G. Kenny D. A. Waters S. M. (2017). Illumina MiSeq 16S amplicon sequence analysis of bovine respiratory disease associated bacteria in lung and mediastinal lymph node tissue. *BMC Vet. Res.* 13:118. 10.1186/s12917-017-1035-2 28464950 PMC5414144

[B32] Kadaikunnan S. Rejiniemon T. Khaled J. M. Alharbi N. S. Mothana R. (2015). In-vitro antibacterial, antifungal, antioxidant and functional properties of *Bacillus amyloliquefaciens*. *Ann. Clin. Microbiol. Antimicrob.* 14:9. 10.1186/s12941-015-0069-1 25858278 PMC4342198

[B33] Kleinhenz M. D. Bumgarner N. R. (2012). Using ^°^brix as an indicator of vegetable quality: an overview of the practice. *Fact Sheet. Agric. Nat. Resourc.* 2:1650.

[B34] Kozich J. J. Westcott S. L. Baxter N. T. Highlander S. K. Schloss P. D. (2013). Development of a dual-index sequencing strategy and curation pipeline for analyzing amplicon sequence data on the MiSeq Illumina sequencing platform. *Appl. Environ. Microbiol.* 79 5112–5120. 10.1128/AEM.01043-13 23793624 PMC3753973

[B35] Li H. Lian B. Ding Y. Nie C. Zhang Q. (2014). Bacterial diversity in the central black component of maotai daqu and its flavor analysis. *Ann. Microbiol.* 64 1659–1669. 10.1007/s13213-014-0809-z

[B36] Li P. Lin W. Liu X. Wang X. Luo L. (2016). Environmental factors affecting microbiota dynamics during traditional solid-state fermentation of Chinese Daqu starter. *Front. Microbiol.* 7:1237. 10.3389/fmicb.2016.01237 27540378 PMC4972817

[B37] Li S. Li P. Feng F. Luo L. X. (2015). Microbial diversity and their roles in the vinegar fermentation process. *Appl. Microbiol. Biotechnol.* 99 4997–5024. 10.1007/s00253-015-6659-1 25971198

[B38] Li S. Li P. Liu X. Luo L. Lin W. (2016). Bacterial dynamics and metabolite changes in solid-state acetic acid fermentation of Shanxi aged vinegar. *Appl. Microbiol. Biotechnol.* 100 4395–4411. 10.1007/s00253-016-7284-3 26754813

[B39] Li X. Yue X. (2016). Analysis of the microbiota in naturally fermented cabbage of northeastern part of China by high-throughput sequencing of the v3-v4 regions of the 16S rRNA Gene. *Int. J. Agric. Biol.* 18 1153–1158. 10.17957/ijab/15.0219 29653435

[B40] Liu H. Sun B. (2018). Effect of fermentation processing on the flavor of Baijiu. *J. Agric. Food Chem.* 66 5425–5432. 10.1021/acs.jafc.8b00692 29751730

[B41] Ljungdahl L. G. (1986). The autotrophic pathway of acetate synthesis in acetogenic bacteria. *Annu. Rev. Microbiol.* 40 415–450. 10.1146/annurev.mi.40.100186.002215 3096193

[B42] Loman N. J. Misra R. V. Dallman T. J. Constantinidou C. Gharbia S. E. Wain J. (2012). Performance comparison of benchtop high-throughput sequencing platforms. *Nat. Biotechnol.* 30 434–439. 10.1038/nbt.2198 22522955

[B43] Lozupone C. Knight R. (2005). UniFrac: a new phylogenetic method for comparing microbial communities. *Appl. Environ. Microbiol.* 71 8228–8235. 10.1128/AEM.71.12.8228-8235.2005 16332807 PMC1317376

[B44] Lozupone C. A. Hamady M. Kelley S. T. Knight R. (2007). Quantitative and qualitative beta diversity measures lead to different insights into factors that structure microbial communities. *Appl. Environ. Microbiol.* 73 1576–1585. 10.1128/AEM.01996-06 17220268 PMC1828774

[B45] Makarova K. Slesarev A. Wolf Y. Sorokin A. Mirkin B. Koonin E. (2006). Comparative genomics of the lactic acid bacteria. *Proc. Natl. Acad. Sci. U.S.A.* 103 15611–15616. 10.1073/pnas.0607117103 17030793 PMC1622870

[B46] Manichanh C. Chapple C. E. Frangeul L. Gloux K. Guigo R. Dore J. (2008). A comparison of random sequence reads versus 16S rDNA sequences for estimating the biodiversity of a metagenomic library. *Nucleic Acids Res.* 36 5180–5188. 10.1093/nar/gkn496 18682527 PMC2532719

[B47] Mardis E. R. (2008). Next-generation DNA sequencing methods. *Annu. Rev. Genomics Hum. Genet.* 9 387–402. 10.1146/annurev.genom.9.081307.164359 18576944

[B48] Moayedi A. Hashemi M. Safari M. (2016). Valorization of tomato waste proteins through production of antioxidant and antibacterial hydrolysates by proteolytic *Bacillus subtilis*: optimization of fermentation conditions. *J. Food Sci. Technol.* 53 391–400. 10.1007/s13197-015-1965-2 26787958 PMC4711411

[B49] Movahedi S. Waites W. (2002). Cold shock response in sporulating *Bacillus subtilis* and its effect on spore heat resistance. *J. Bacteriol.* 184 5275–5281. 10.1128/JB.184.19.5275-5281.2002 12218012 PMC135340

[B50] Nelson M. C. Morrison H. G. Benjamino J. Grim S. L. Graf J. (2014). Analysis, optimization and verification of Illumina-generated 16S rRNA gene amplicon surveys. *PLoS One* 9:e94249. 10.1371/journal.pone.0094249 24722003 PMC3983156

[B51] Oyewole O. B. (1997). Lactic fermented foods in Africa and their benefits. *Food Control.* 8 289–297. 10.1016/s0956-7135(97)00075-3

[B52] Pang X. N. Han B. Z. Huang X. N. Zhang X. Hou L. F. Cao M. (2018). Effect of the environment microbiota on the flavour of light-flavour Baijiu during spontaneous fermentation. *Sci. Rep.* 8:3396. 10.1038/s41598-018-21814-y 29467508 PMC5821866

[B53] Pang X. N. Huang X. N. Chen J. Y. Yu H. X. Wang X. Y. Han B. Z. (2020). Exploring the diversity and role of microbiota during material pretreatment of light-flavor Baijiu. *Food Microbiol.* 91 103514. 10.1016/j.fm.2020.103514 32539964

[B54] Peris M. Escuder-Gilabert L. (2016). Electronic noses and tongues to assess food authenticity and adulteration. *Trends Food Sci. Technol.* 58 40–54. 10.1016/j.tifs.2016.10.014

[B55] Piard J. C. Desmazeaud M. (1992). Inhibiting factors produced by lactic acid bacteria. 2. Bacteriocins and other antibacterial substances. *Le Lait* 72 113–142. 10.1051/lait:199229

[B56] Polka J. Rebecchi A. Pisacane V. Morelli L. Puglisi E. (2015). Bacterial diversity in typical Italian salami at different ripening stages as revealed by high-throughput sequencing of 16S rRNA amplicons. *Food Microbiol.* 46 342–356. 10.1016/j.fm.2014.08.023 25475305

[B57] Robichaud J. L. Noble A. C. (1990). Astringency and bitterness of selected phenolics in wine. *J. Sci. Food Agric.* 53 343–353. 10.1002/jsfa.2740530307

[B58] Rodriguez-Mendez M. L. Apetrei C. Gay M. Medina-Plaza C. de Saja J. A. Vidal S. (2014). Evaluation of oxygen exposure levels and polyphenolic content of red wines using an electronic panel formed by an electronic nose and an electronic tongue. *Food Chem.* 155 91–97. 10.1016/j.foodchem.2014.01.021 24594159

[B59] Roh S. W. Kim K. H. Nam Y. D. Chang H. W. Park E. J. Bae J. W. (2010). Investigation of archaeal and bacterial diversity in fermented seafood using barcoded pyrosequencing. *ISME J* 4 1–16. 10.1038/ismej.2009.83 19587773

[B60] Schuchmann K. Muller V. (2014). Autotrophy at the thermodynamic limit of life: a model for energy conservation in acetogenic bacteria. *Nat. Rev. Microbiol.* 12 809–821. 10.1038/nrmicro3365 25383604

[B61] Sengun I. Y. Karabiyikli S. (2011). Importance of *acetic acid bacteria* in food industry. *Food Control.* 22 647–656. 10.1016/j.foodcont.2010.11.008

[B62] Simpson E. H. (1949). Measurement of diversity. *Nature* 163 688–688. 10.1038/163688a0

[B63] Song Z. Du H. Zhang Y. Xu Y. (2017). Unraveling core functional microbiota in traditional solid-state fermentation by high-throughput amplicons and metatranscriptomics sequencing. *Front. Microbiol.* 8:1294. 10.3389/fmicb.2017.01294 28769888 PMC5509801

[B64] Soomro A. H. Masud T. Anwaar K. (2001). Role of lactic acid bacteria (LAB) in food preservation and human health – a review. *Pak. J. Nutr.* 1 20–24. 10.3923/pjn.2002.20.24

[B65] Su Y. Yang L. Hui L. Yuan-Yuan G. Ming-Juan Z. Chun-Hui X. (2015). Bacterial communities during the process of high-temperature Daqu production of roasted sesame-like flavour liquor. *J. Inst. Brew..* 121 440–448. 10.1002/jib.235

[B66] Sun X. Lyu G. Luan Y. Zhao Z. Yang H. Su D. (2018). Analyses of microbial community of naturally homemade soybean pastes in Liaoning Province of China by Illumina Miseq Sequencing. *Food Res. Int.* 111 50–57. 10.1016/j.foodres.2018.05.006 30007713

[B67] Tannock G. W. (2004). A special fondness for lactobacilli. *Appl. Environ. Microbiol.* 70 3189–3194. 10.1128/AEM.70.6.3189-3194.2004 15184111 PMC427720

[B68] Vlasov Y. Legin A. Rudnitskaya A. Di Natale C. D’Amico A. (2005). Nonspecific sensor arrays (“electronic tongue”) for chemical analysis of liquids (IUPAC technical report). *Pure Appl. Chem.* 77 1965–1983. 10.1351/pac200577111965

[B69] Wang C. L. Shi D. J. Gong G. L. (2008). Microorganisms in Daqu: a starter culture of Chinese Maotai-flavor liquor. *World J. Microbiol. Biotechnol.* 24 2183–2190. 10.1007/s11274-008-9728-0

[B70] Wang H. Xu Y. (2019). Microbial succession and metabolite changes during the fermentation of Chinese light aroma-style liquor. *J. Inst. Brew.* 125 162–170. 10.1002/jib.544

[B71] Wang H. Y. Gao Y. B. Fan Q. W. Xu Y. (2011). Characterization and comparison of microbial community of different typical Chinese liquor Daqus by PCR-DGGE. *Lett. Appl. Microbiol.* 53 134–140. 10.1111/j.1472-765X.2011.03076.x 21554340

[B72] Wang H. Y. Xu Y. (2015). Effect of temperature on microbial composition of starter culture for Chinese light aroma style liquor fermentation. *Lett. Appl. Microbiol.* 60 85–91. 10.1111/lam.12344 25346191

[B73] Wang H. Y. Zhang X. J. Zhao L. P. Xu Y. (2008). Analysis and comparison of the bacterial community in fermented grains during the fermentation for two different styles of Chinese liquor. *J. Ind. Microbiol. Biotechnol.* 35 603–609. 10.1007/s10295-008-0323-z 18317829

[B74] Wang P. Mao J. Meng X. Li X. Liu Y. Feng H. (2014). Changes in flavour characteristics and bacterial diversity during the traditional fermentation of Chinese rice wines from Shaoxing region. *Food Control.* 44 58–63. 10.1016/j.foodcont.2014.03.018

[B75] Wang X. Du H. Zhang Y. Xu Y. (2018). Environmental microbiota drives microbial succession and metabolic profiles during Chinese liquor fermentation. *Appl. Environ. Microbiol.* 84 e2369–e2317. 10.1128/AEM.02369-17 29196296 PMC5795089

[B76] Wang X. C. Guo K. L. (2011). Research on aerial bacterial floras in moutai distillery. *Liq. Making Sci. Technol.* 9 29–31. 10.13746/j.njkj.2011.09.038

[B77] Wang Z. M. Lu Z. M. Shi J. S. Xu Z. H. (2016). Exploring flavour-producing core microbiota in multispecies solid-state fermentation of traditional Chinese vinegar. *Sci. Rep.* 6:26818. 10.1038/srep26818 27241188 PMC4886211

[B78] Whittaker R. H. (1965). Dominance and diversity in land plant communities: numerical relations of species express the importance of competition in community function and evolution. *Science* 147 250–260. 10.1126/science.147.3655.250 17788203

[B79] Wijaya D. R. Sarno R. Zulaika E. Sabila S. I. (2017). Development of mobile electronic nose for beef quality monitoring. *Proc. Comp. Sci.* 124 728–735. 10.1016/j.procs.2017.12.211

[B80] Wu Q. Zhu W. Wang W. Xu Y. (2015). Effect of yeast species on the terpenoids profile of Chinese light-style liquor. *Food Chem.* 168 390–395. 10.1016/j.foodchem.2014.07.069 25172725

[B81] Xiao C. Lu Z. M. Zhang X. J. Wang S. T. Ao L. Shen C. H. (2017). Bio-heat is a key environmental driver shaping the microbial community of medium-temperature Daqu. *Appl. Environ. Microbiol.* 83 e1550–e1517. 10.1128/AEM.01550-17 28970223 PMC5691423

[B82] Xiong Y. Zhang P. Warner R. D. Fang Z. (2019). Sorghum grain: from genotype, nutrition, and phenolic profile to its health benefits and food applications. *Compr. Rev. Food Sci. Food Saf.* 18 2025–2046. 10.1111/1541-4337.12506 33336966

[B83] Xu Y. Wang D. Fan W. L. Mu X. Q. Chen J. (2010). Traditional Chinese biotechnology. *Adv. Biochem. Eng. Biotechnol.* 122 189–233. 10.1007/10_2008_36 19888561

[B84] Yan Z. Zheng X.-W. Chen J.-Y. Han J.-S. Han B.-Z. (2013a). Effect of different Bacillusstrains on the profile of organic acids in a liquid culture of Daqu. *J. Inst. Brew.* 119 78–83. 10.1002/jib.58

[B85] Yan Z. Zheng X. W. Han B. Z. Han J. S. Nout M. J. Chen J. Y. (2013b). Monitoring the ecology of Bacillus during Daqu incubation, a fermentation starter, using culture-dependent and culture-independent methods. *J. Microbiol. Biotechnol.* 23 614–622. 10.4014/jmb.1211.11065 23648849

[B86] Yang F. Chen L. Liu Y. Li J. Wang L. Chen J. (2019). Identification of microorganisms producing lactic acid during solid-state fermentation of Maotai flavour liquor. *J. Inst. Brew.* 125 171–177. 10.1002/jib.537

[B87] Ye L. Shao M. F. Zhang T. Tong A. H. Lok S. (2011). Analysis of the bacterial community in a laboratory-scale nitrification reactor and a wastewater treatment plant by 454-pyrosequencing. *Water Res.* 45 4390–4398. 10.1016/j.watres.2011.05.028 21705039

[B88] Yong H. S. Song S. L. Chua K. O. Lim P. E. (2017). High diversity of bacterial communities in developmental stages of *Bactrocera carambolae* (Insecta: Tephritidae) revealed by illumina MISEQ sequencing of 16S rRNA Gene. *Curr. Microbiol.* 74 1076–1082. 10.1007/s00284-017-1287-x 28642971

[B89] Zhang J. Wang X. Huo D. Li W. Hu Q. Xu C. (2016). Metagenomic approach reveals microbial diversity and predictive microbial metabolic pathways in Yucha, a traditional Li fermented food. *Sci. Rep.* 6:32524. 10.1038/srep32524 27578483 PMC5006176

[B90] Zhang L. Wu C. Ding X. Zheng J. Zhou R. (2014). Characterisation of microbial communities in Chinese liquor fermentation starters Daqu using nested PCR-DGGE. *World J. Microbiol. Biotechnol.* 30 3055–3063. 10.1007/s11274-014-1732-y 25193747

[B91] Zhang W. X. Qiao Z. W. Shigematsu T. Tang Y. Q. Hu C. Morimura S. (2005). Analysis of the bacterial community in Zaopei during production of Chinese Luzhou-flavor liquor. *J. Inst. Brew.* 111 215–222. 10.1002/j.2050-0416.2005.tb00669.x

[B92] Zhang X. Zhao J. Du X. (2014). Barcoded pyrosequencing analysis of the bacterial community of Daqu for light-flavour Chinese liquor. *Lett. Appl. Microbiol.* 58 549–555. 10.1111/lam.12225 24471485

[B93] Zhao X. Wang Y. Cai W. Yang M. Zhong X. Guo Z. (2020). High-throughput Sequencing-based analysis of microbial diversity in rice Wine Koji from different areas. *Curr. Microbiol.* 77 882–889. 10.1007/s00284-020-01877-9 31950235

[B94] ZhengX.-W.HanB.-Z. (2016). Baijiu ( 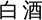 ), Chinese liquor: history, classification and manufacture. *J. Ethn. Foods* 3 19–25. 10.1016/j.jef.2016.03.001

[B95] Zheng X. W. Tabrizi M. R. Nout M. J. R. Han B. Z. (2011). Daqu - a traditional Chinese liquor fermentation starter. *J. Inst. Brew.* 117 82–90. 10.1002/j.2050-0416.2011.tb00447.x

[B96] Zheng X. W. Yan Z. Han B. Z. Zwietering M. H. Samson R. A. Boekhout T. (2012). Complex microbiota of a Chinese “Fen” liquor fermentation starter (Fen-Daqu), revealed by culture-dependent and culture-independent methods. *Food Microbiol.* 31 293–300. 10.1016/j.fm.2012.03.008 22608236

[B97] Zheng X. W. Yan Z. Nout M. J. Smid E. J. Zwietering M. H. Boekhout T. (2014). Microbiota dynamics related to environmental conditions during the fermentative production of Fen-Daqu, a Chinese industrial fermentation starter. *Int. J. Food Microbiol.* 18 57–62. 10.1016/j.ijfoodmicro.2014.05.008 24863368

