# Supplementary figures and images for: Bacterial Diversity, Organic Acid, and Flavor Analysis of Dacha and Ercha Fermented Grains of Fen Flavor Baijiu
Source: Front Microbiol. 2022 Jan 4;12:769290. doi: 10.3389/fmicb.2021.769290 (PMC8765705; doi:10.3389/fmicb.2021.769290)

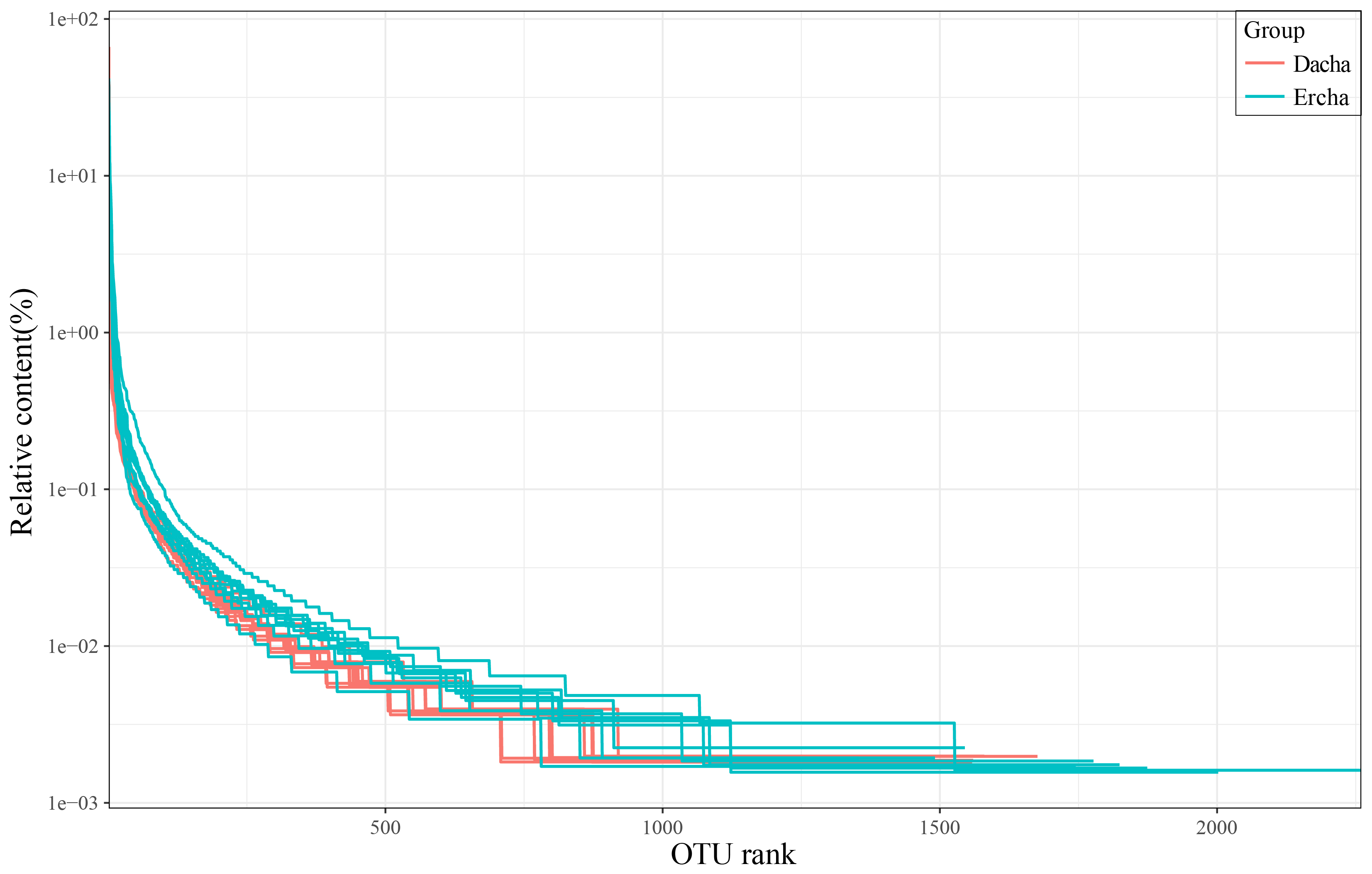

Supplement: Supplementary Figure 1 — Rank-abundance curve. [file Image_1.JPEG]

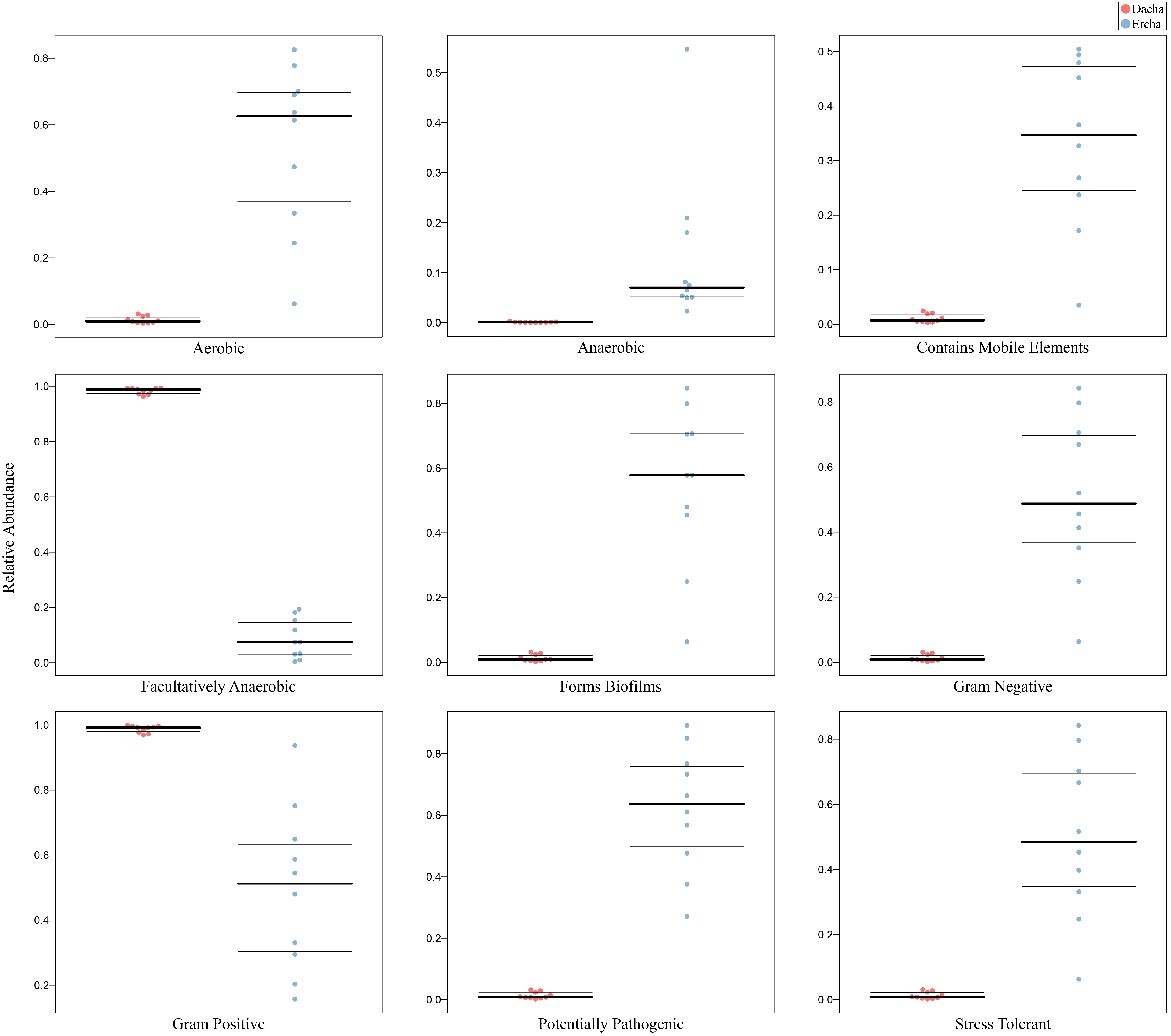

Supplement: Supplementary Figure 2 — Comparative analysis of bacterial phenotypic results. [file Image_2.JPEG]
